# Stimulation of Locus Ceruleus Inputs to the Prelimbic Cortex in Mice Induces Cell Type-Specific Expression of the *Apoe* Gene

**DOI:** 10.1523/ENEURO.0328-24.2024

**Published:** 2024-12-10

**Authors:** Genevieve E. Craig, Lizbeth Ramos, Samuel R. Essig, Nicholas J. Eagles, Andrew E. Jaffe, Keri Martinowich, Henry L. Hallock

**Affiliations:** ^1^Neuroscience Program, Lafayette College, Easton, Pennsylvania 18042; ^2^Lieber Institute for Brain Development, Johns Hopkins Medical Campus, Baltimore, Maryland 21205; ^3^Departments of Psychiatry and Behavioral Sciences, Johns Hopkins School of Medicine, Baltimore, Maryland 21205; ^4^Neuroscience, Johns Hopkins School of Medicine, Baltimore, Maryland 21205; ^5^The Kavli Neuroscience Discovery Institute, Johns Hopkins University, Baltimore, Maryland 21205

## Abstract

The medial frontal cortex (mFC) and locus ceruleus (LC) are two brain areas that have been implicated in a range of cognitive phenomena, such as attention, memory, and decision-making. Regulators of these brain regions at the molecular level are not well understood but might help to elucidate underlying mechanisms of disorders that present with deficits in these cognitive domains. To probe this, we used chemogenetic stimulation of neurons in the LC with axonal projections to the prelimbic subregion (PrL) of the mFC and subsequent bulk RNA sequencing from the mouse PrL. We found that stimulation of this circuit caused an increase in transcription of a host of genes, including the *Apoe* gene. To investigate cell type-specific expression of *Apoe* in the PrL, we used a dual-virus approach to express either the excitatory DREADD receptor hM3Dq in LC neurons with projections to the PrL or a control virus and found that increases in *Apoe* expression in the PrL following depolarization of LC inputs is enriched in GABAergic neurons in a sex-dependent manner. The results of these experiments yield insights into how *Apoe* expression affects function in a cortical microcircuit that is important for attention, memory, and decision-making and point to interneuron-specific expression of *Apoe* as a potential biomarker for circuit function in disorders such as attention-deficit hyperactivity disorder, schizophrenia, and Alzheimer's disease.

## Significance Statement

Identifying patterns of gene expression in specific brain circuits is an important first step toward developing treatments for cognitive and behavioral symptoms that rely on those circuits. In this paper, we describe a transcriptome-scale motif in one such circuit—neurons in the locus ceruleus that project to the prelimbic subregion. This circuit has been implicated in attention, memory, and decision-making, and deficits in these cognitive domains are common across many neuropsychiatric disorders. We further explored one of the top differentially expressed genes, *Apoe*, to identify how it is expressed in distinct cell types following stimulation of this circuit, paving the way for spatially and genetically specific targeting of this gene in disorders that feature dysfunction in this circuit.

## Introduction

The anterior cingulate cortex (ACC), located on the medial surface of the frontal lobes in humans, is heavily involved in a variety of cognitive domains, including attention (Davis et al., 2000), memory (Kozlovskiy et al., 2012), and decision-making (Walton and Mars, 2007). For example, the ACC is highly active during sustained attention (MacDonald et al., 2000), as well as conflict monitoring, which is the allocation of attention based on conflicting signals (Carter et al., 1998). ACC activity is also correlated with better performance on tasks that measure working memory (Lenartowicz and McIntosh, 2005) and remote memory (Petersen et al., 1988). ACC activity during these tasks is likely influenced by neuromodulatory systems, such as the locus ceruleus (LC), which supplies cortical norepinephrine (NE) and is reciprocally connected to the ACC in primates (Joshi and Gold, 2022) and rodents (Jodoj et al., 1998; Pudovkina et al., 2001). LC-NE activation influences ACC activity patterns and affects both attention (Dahl et al., 2020) and memory (Murchison et al., 2004; Bahtiyar et al., 2020), implicating the LC as a driver of cortical function in attention and memory-guided behavior. Deficits in ACC and LC function are also present in disorders in which attention and memory deficits are a common cognitive symptom, such as schizophrenia, attention-deficit hyperactivity disorder (ADHD), and major depressive disorder (MDD; Kerns et al., 2005; del Cerro et al., 2020; Shirama et al., 2020), highlighting the importance of understanding how the LC and ACC functionally interact with one another.

Translating research on the primate ACC to rodents has been difficult, in part because identifying regions in the rodent that are homologous to primate cortical areas is not straightforward. Recent research has provided strong evidence that the area commonly referred to as the medial prefrontal cortex (mPFC) in rodents is actually more anatomically homologous with the human cingulate cortex (Laubach et al., 2018; van Heukelum et al., 2020), providing an avenue for comparison of ACC activity between species. In support of this, many studies in rodents have shown that activity in the prelimbic subregion (PrL) of the rodent mPC is strongly related to attention (Birrell and Brown, 2000; Passetti et al., 2002; Fisher et al., 2020) and memory (Brito and Brito, 1990; Delatour and Gisquet-Verrier, 1996; Hallock et al., 2016; Ye et al., 2017). Furthermore, catecholamine signaling in the rodent PrL is involved in attention (Berridge and Spencer, 2016) and memory (Tronel et al., 2004), and drugs that modulate catecholaminergic function often affect memory and attention in rodents (Sherrill et al., 2013; Mar et al., 2017; Caballero-Puntiverio et al., 2019; McDonald et al., 2021), suggesting that brain areas like the LC drive function in cortical areas to promote rodent cognition as well. The PrL and LC functionally interact during rodent versions of attention (Bouret and Sara, 2004; Newman et al., 2008; Hallock et al., 2024) and memory tasks (Giustino et al., 2019), further corroborating the idea that the LC and PrL work together during attention and memory-guided behavior across mammalian species.

Although specific brain circuits, like the one between the LC and PrL, have been implicated in a range of cognitive processes, little research has been done to understand the molecular drivers of function in these circuits. Gene expression in cortical tissue is heterogeneous between brain regions and cell types in both humans (Darmanis et al., 2015; Maynard et al., 2021) and rodents (Zeisel et al., 2015), and these patterns of gene expression change in response to behavioral experience (Cho et al., 2016; Mukherjee et al., 2018), suggesting that unique cell type-specific patterns of gene expression could be used as markers of circuit function. These results also raise the possibility that cells embedded in specific circuits could be noninvasively accessed by manipulating genes selectively expressed in those cells, paving the way for gene-targeted treatments for cognitive symptoms in neuropsychiatric disorders, such as deficits in attention, memory, and decision-making. In order to uncover gene expression motifs in one such circuit, we used chemogenetic targeting to activate neurons in the LC that send direct axonal projections to the PrL in mice. We subsequently used bulk RNA sequencing and single-molecule in situ hybridization to look at cell type-specific transcription in the PrL in response to stimulation of LC inputs. We found that, among other genes, the gene encoding for the apolipoprotein E protein (*Apoe*) was enriched in PrL tissue following circuit activation. This increase in *Apoe* transcription was driven by expression in GABAergic neurons, and this effect was more robust in female mice.

## Materials and Methods

### Subjects

For the RNA sequencing experiment, we used a cohort of eight male (four experimental and four control) wild-type c57bl/6j mice (Jackson strain 000664). For RNAscope experiments in the PrL, we used a cohort of 12 female (6 experimental and 6 control) and 12 male (6 experimental and 6 control) wild-type c57bl/6j mice (24 total mice). Mice were group housed (3–5 animals per cage) with *ad libitum* access to both food and water. The colony room was temperature and humidity controlled on a 12 h light/dark cycle. All experiments were performed during the light cycle. At time of surgery, animals were roughly 90–120 d of age. All procedures were in accordance with the Institutional Animal Care and Use Committees of The Lieber Institute for Brain Development (mice used in the RNA sequencing experiment) and Lafayette College (mice used in RNAscope experiments).

### Surgical and extraction procedures

**Figure 1.**
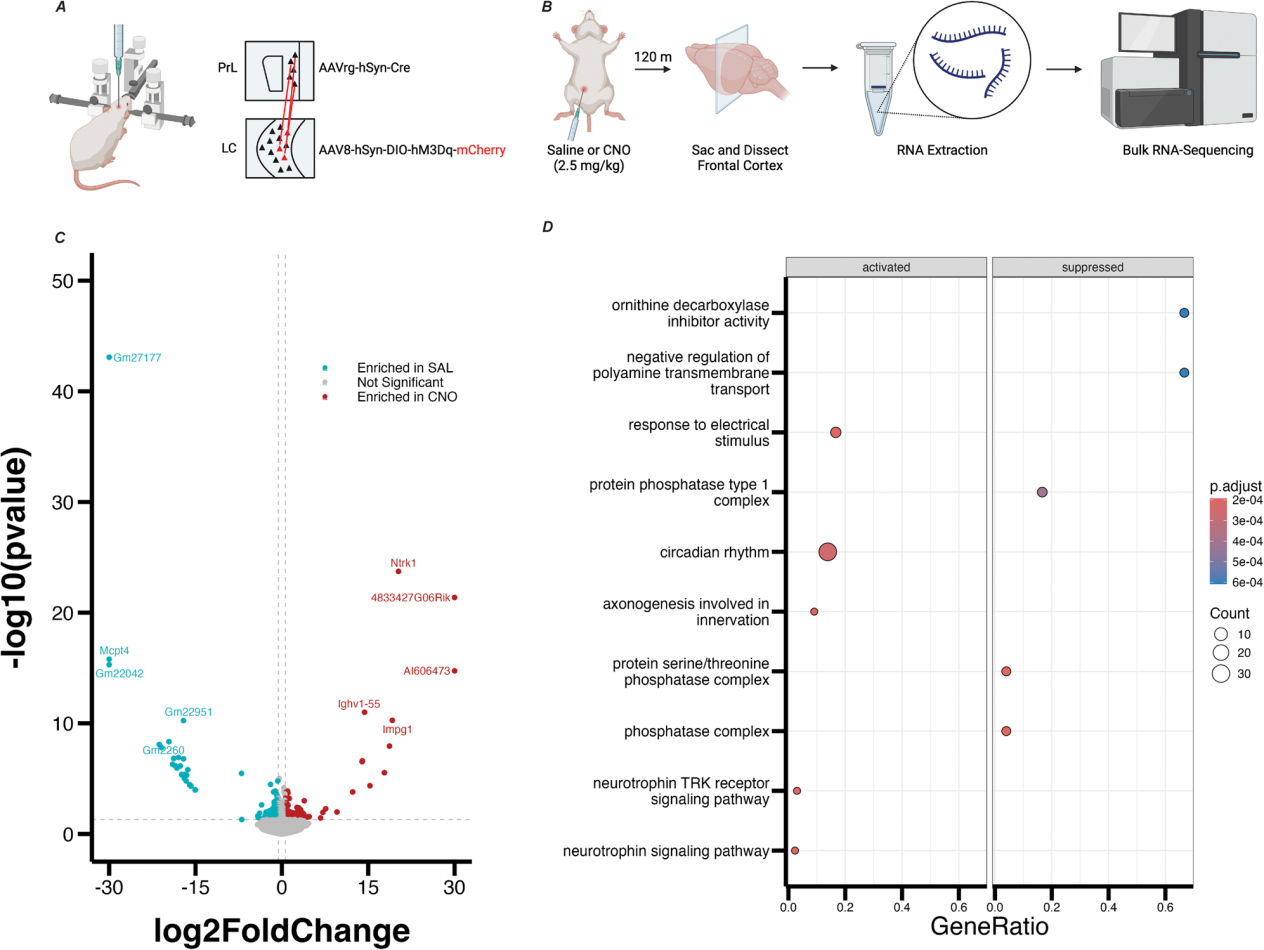
RNA sequencing reveals enrichment of genes in PrL tissue following stimulation of LC inputs. ***A***, Schematic showing viral injection strategy for chemogenetic activation of prelimbic cortex (PrL)-projecting neurons in the locus ceruleus (LC). ***B***, Experimental design for identification of enriched gene sets in the PrL following chemogenetic activation of PrL-projecting LC neurons. ***C***, Volcano plot showing genes that were enriched in PrL tissue in the experimental (CNO-receiving) group of mice (red dots) and genes that were enriched in PrL tissue in the control (saline-receiving) group of mice (blue dots) based on differential expression analysis. The top 10 differentially expressed genes (based on FDR-adjusted *p* value) are labeled. ***D***, Dot plot showing gene ontology terms that contained differentially expressed gene sets for experimental mice (left column) and control mice (right column). The top 10 gene ontology terms by FDR-adjusted *p* value are listed on the *y*-axis.

**Figure 2.**
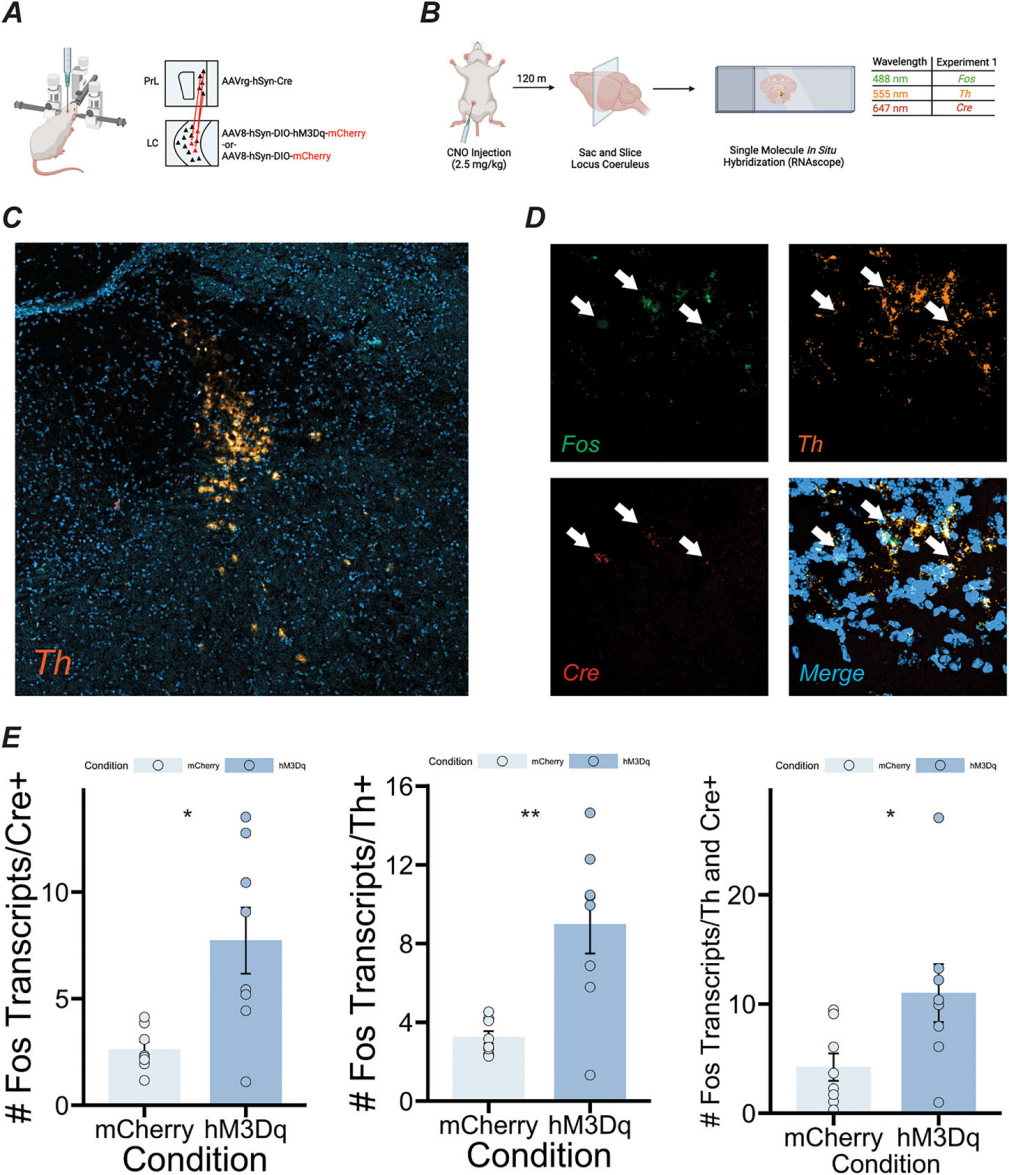
Verification of DREADD-mediated activation of LC neurons. ***A***, Schematic showing viral injection strategy for activation of PrL-projecting neurons in the LC for RNAscope experiments. ***B***, Experimental design. ***C***, Confocal image showing tyrosine hydroxylase (Th) expression in the locus ceruleus (LC). ***D***, Confocal image at 40× magnification showing expression of Fos, Th, and Cre in LC tissue. ***E***, In mice that had the DREADD receptor, Fos expression was increased in Cre+ cells (left panel—*t*_(7.786)_ = 3.2078, *p* = 0.01292), Th + cells (middle panel—*t*_(7.6345)_ = 3.8082, *p* = 0.005647), and cells that were both Th and Cre+ (right panel—*t*_(9.9693)_ = 2.3047, *p* = 0.04398) compared with mCherry controls. Error bars = SEM.

For all experiments, mice were anesthetized with isoflurane (1–2.5% oxygen) and then placed into a stereotaxic frame (Kopf Instruments). An incision was made along the midline of the scalp, the skull was leveled, and bregma was identified. Holes were drilled in the skull above the target brain regions (LC, −5.4 mm AP from the bregma, ±0.9 mm ML from the midline; PrL, +1.7 mm AP from the bregma, ±0.3 mm ML from the midline), and an automated infusion pump (World Precision Instruments) was used to inject the viruses at 3 nl/s for a total volume of 600 nl/hemisphere in the PrL (1.7 mm ventral to the surface of the brain) and 300 nl/hemisphere in the LC (3.0 mm ventral to the surface of the brain). Measurements for these brain regions were obtained from the Paxinos and Franklin mouse brain atlas (Paxinos and Franklin, 2019). For the RNA sequencing experiment, a retrograde virus encoding Cre-recombinase (AAVrg-hSyn-Cre; Addgene, catalog #105553-AAVrg) was injected into the PrL, and a virus coding for the Cre-dependent expression of an excitatory DREADD receptor (AAV8-hSyn-DIO-hM3Dq-mCherry; Addgene, catalog # 44361-AAV8) was injected into the LC (Fig. 1*A*). For RNAscope experiments, AAVrg-hSyn-Cre was injected into the PrL, and either a virus coding for the Cre-dependent expression of an excitatory DREADD receptor (AAV8-hSyn-DIO-hM3Dq-mCherry; experimental group) or a control virus (AAV8-hSyn-DIO-mCherry; control group; Addgene, catalog #114472-AAV8) was injected into the LC (Fig. 2*A*). For the RNA sequencing experiment, we waited 4–5 weeks for the virus to infect a sufficient number of cells and then gave intraperitoneal injections of either clozapine-*N*-oxide (CNO; 2.5 mg/kg; experimental group; Tocris Bioscience, catalog #4936) or vehicle [filtered 1× phosphate-buffered saline (PBS); control group] into the mice. Vehicle injections were matched to the volume of CNO injections in experimental mice. Because CNO exerts its peak effects after ∼30 min, and peak expression of many immediate early genes (IEGs) is typically seen ∼90 min after stimulation (Barry and Commins, 2017), we killed the mice 120 min following injections. Following cervical dislocation, the brains of the mice were extracted, the medial wall of the PrL from both hemispheres was dissected with a brain block and razor blades on wet ice, and each hemisphere was flash-frozen in 2-methylbutane and stored in a 1.5 ml Eppendorf centrifuge tube at −80°C. For RNAscope experiments, we again waited 4–5 weeks for the virus to infect a sufficient number of cells, gave all mice CNO injections (2.5 mg/kg, i.p.), and killed the mice 120 min following injections. We then extracted the brains, flash-froze them in 2-methylbutane, and stored them at −80°C.

10.1523/ENEURO.0328-24.2024.f1-1 Figure 1-1 Table showing log fold changes, p-values, and FDR-adjusted p-values for all differentially-expressed genes in the bulk RNA-sequencing experiment. Download Figure 1-1, XLS file.

10.1523/ENEURO.0328-24.2024.f1-2 Figure 1-2 Table showing p-values, FDR-adjusted-values, and core genes (in ENSEMBL notation) for all gene ontology (GO) terms from the bulk RNA-sequencing experiment. Download Figure 1-2, XLS file.

### Bulk RNA sequencing

Total RNA was isolated and extracted from the tissue using TRIzol (Life Technologies), purified using RNeasy minicolumns (Qiagen; catalog #74104), and quantified using a NanoDrop spectrophotometer (Agilent Technologies). The Nextera XT DNA Library Preparation Kit was used to generate sequencing libraries according to manufacturer instructions, and samples were sequenced on a HiSeq 2000 (Illumina). Reads were aligned to the mm10 genome using the HISAT2 splice-aware aligner (D. Kim et al., 2015), and alignments overlapping genes were counted using featureCounts version 1.5.0-p3 relative to Gencode version M11. Differential expression analyses were performed on gene counts using the voom approach (Law et al., 2014) in the limma R/Bioconductor package (Ritchie et al., 2015) using weighted trimmed means normalization factors with condition (CNO vs saline) as the main outcome of interest and adjusting for the exonic mapping rate. Multiple testing correction was performed using the Benjamini–Hochberg approach to control for the false discovery rate (FDR). Gene ontology (GO) analyses were performed using ENSEMBL gene IDs with the clusterProfiler R Bioconductor package (Ashburner et al., 2000; Yu et al., 2012).

### Single-molecule fluorescent in situ hybridization

To perform single-molecule in situ hybridization (RNAscope) on LC and PrL tissue, we took coronal sections of the PrL and LC (16 μm) on a cryostat (Leica), mounted them onto slides, and performed the RNAscope protocol using the fluorescent multiplex V2 kit from ACDBio (catalog #323110). Specifically, tissue sections were briefly fixed with a 10% neutral buffered formalin solution at room temperature and subjected to serious dehydration with ethanol. We then pretreated the sections with protease IV and hydrogen peroxide and incubated the slides at 40°C with a combination of three probes. For PrL tissue, these probes were as follows: channel 1, *Gfap*; channel 2, *Rbfox3*; channel 3, *Apoe* for Experiment 1; channel 1, *Slc17a7*; channel 2, *Gad1*; channel 3, *Apoe* for Experiment 2; and channel 1, *Sst*; channel 2, *Pvalb*; channel 3, *Apoe* for Experiment 3. For LC tissue, these probes were as follows: channel 1, *Fos*; channel 2, *Th*; channel 3, *Cre*. After incubation, we applied amplification buffers for each channel and opal dyes (520 nm for channel 1; 570 nm for channel 2; 690 nm for channel 3; Akoya Biosciences, catalog #FP1487001KT, FP1488001KT, and FP1497001KT) in order to fluorescently label each transcript. Lastly, we stained the sections with DAPI to demarcate the nuclei of the cells. We then took *z*-stacked images of the PrL (four sections per slide, one image per section, four images per mouse total) and LC slides (one section per slide, one image per section, one image per mouse total) using a Zeiss LSM800 confocal microscope. For analysis, we used a MATLAB program to quantify transcript expression in each image (*dotdotdot*; Maynard et al., 2020). Specifically, we used the “CellSegm” toolbox to perform nuclear segmentation in *x*, *y*, and *z*-dimensions to define regions of interest (ROIs) based on DAPI expression and watershed analysis to identify distinct transcripts (individual dots) in each of the three microscope channels corresponding to an opal dye. We then colocalized each identified transcript with an identified nucleus (ROI). Transcripts that were not classified by the program as being colocalized with a nucleus were not used for analysis. Background noise, which could result from bleed-through from adjacent wavelengths in the gene channels, was eliminated using the “imhmin” function. This function suppresses all of the minima in the grayscale image whose depth is less than the standard deviation of the image. We used a cutoff of five transcripts to categorize a cell as either *Rbfox3* or *Gfap*-expressing, *Gad1* or *Slc17a7*-expressing, and *Pvalb* or *Sst*-expressing, respectively.

### Statistical analysis

For RNAscope results, we used multiple two-way analyses of variance (ANOVAs) to compare gene expression between sex and experimental condition (DREADD vs mCherry controls). We also used Welch's two-sample *t* tests to compare gene expression in the LC between DREADD and mCherry controls. An alpha level of 0.05 was used to determine statistical significance. All tests were performed with the R programming language (“aov” and “*t*.test” functions).

## Results

### RNA sequencing

We identified 96 genes that were differentially expressed in PrL tissue between our CNO and saline groups (genes that had an FDR-adjusted *p* value < 0.05). Of these genes, 82 were enriched in the saline group, and 14 were enriched in the CNO group (Fig. 1*C*). Gene ontology (GO) analysis revealed that genes enriched in the CNO group are involved in circadian rhythms, axonogenesis, and neurotrophin signaling, suggesting that stimulation of LC inputs to the PrL induces signaling pathways involved in plasticity (Fig. 1*D*). Of the genes that were enriched in the CNO group, the *Apoe* gene (log2FoldChange = 0.34, FDR-adjusted *p* value = 0.0342) stood out due to its involvement in attention and memory in humans (Parasuraman et al., 2002; Jochemsen et al., 2012; Rusted et al., 2013). Although there were other genes that were more significantly upregulated in our geneset, none of these genes had obvious links to cognition or disorders of the nervous system, making *Apoe* an attractive candidate to study further. A complete list of differentially expressed genes is available in the extended data (Extended Data Fig. 1-1).

### Single-molecule in situ hybridization

In order to verify our RNA sequencing results, and determine cell type-specific expression of *Apoe* in PrL tissue following depolarization of LC inputs, we replicated our RNA sequencing experiment with several important changes. First, we used both male and female mice in order to determine whether sex differences in *Apoe* expression levels might be present, as previous research has demonstrated sex differences in mouse LC anatomy and function (Bangasser et al., 2011, 2016). Second, we employed an experimental design in which all mice received CNO injections, as the presence of CNO alone could have accounted for the increase in *Apoe* transcription that we observed in our RNA sequencing data.

Firstly, to verify that our viral targeting strategy increased neuronal activity in LC neurons, we took coronal sections of the LC and quantified *Fos* expression in neurons expressing tyrosine hydroxylase (*Th*-expressing neurons) and neurons expressing Cre-recombinase (*Cre*-expressing neurons; Fig. 2*C*,*D*). Due to the difficulties inherent in slicing LC tissue, we were only able to obtain usable slices from 16 total mice (12 male, 6 in the experimental group, 6 in the control group; 4 female, 2 in the experimental group, 2 in the control group). We found evidence that *Th*- and *Cre*-expressing neurons in the LC were robustly activated following CNO injections in the experimental group, as *Fos* transcription was increased in *Cre*-expressing (*t*_(7.786)_ = 3.2078; *p *= 0.01292), *Th*-expressing (*t*_(7.6345)_ = 3.8082; *p *= 0.005647), and both *Cre*- and *Th*-expressing (*t*_(9.9693)_ = 2.3047; *p *= 0.04398) neurons in the LC of experimental mice compared with mice in the control group (Welch's *t* tests; Fig. 2*E*). Although the small number of female mice in our study precluded analysis of sex differences for this particular experiment, when data were graphed separately for males and females, we saw that *Fos* expression was increased in LC neurons of the experimental group in both sexes.

**Figure 3.**
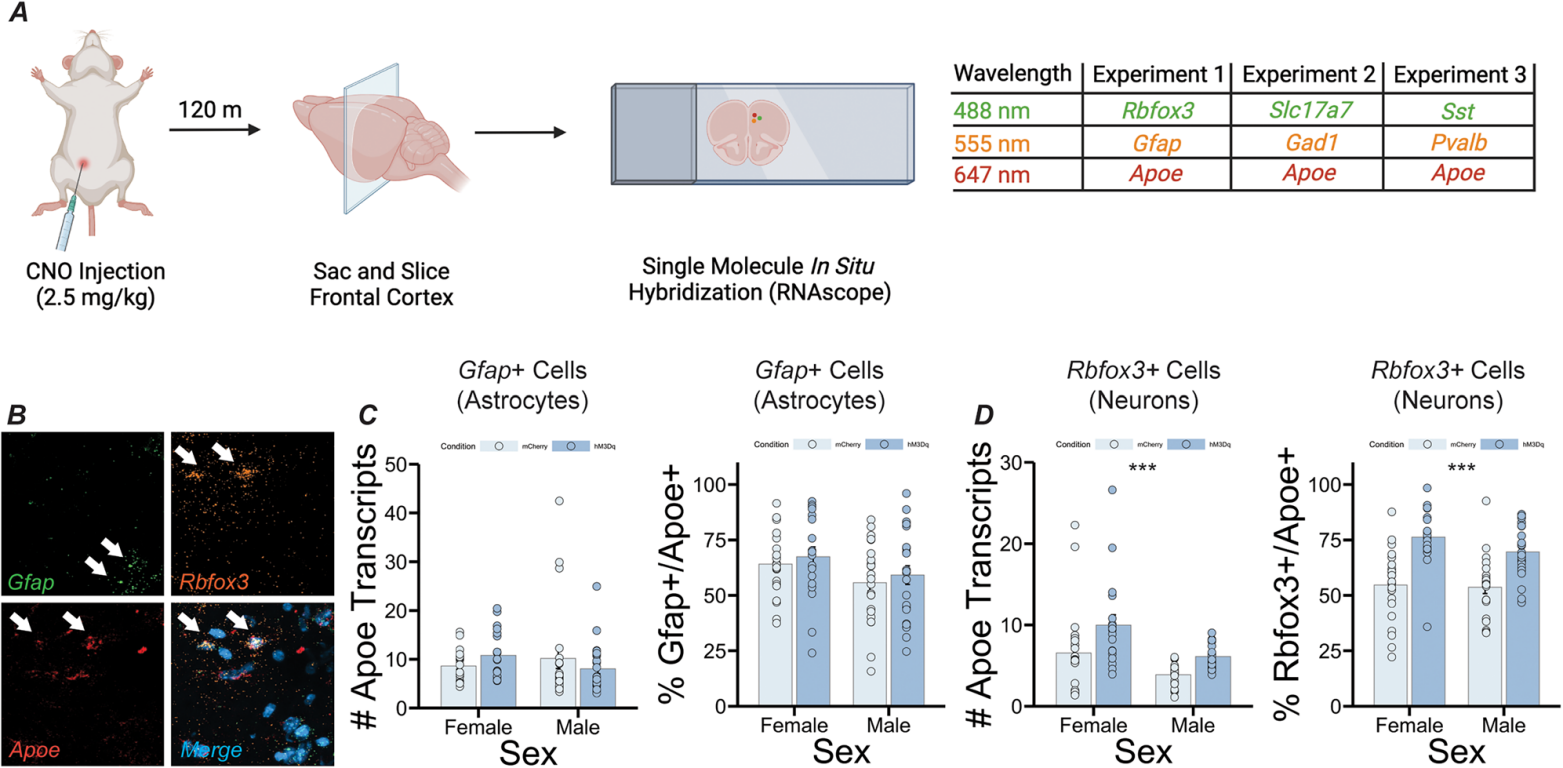
Enrichment of *Apoe* in PrL neurons following stimulation of LC inputs. ***A***, Experimental design. ***B***, Confocal image showing expression of *Gfap*, *Rbfox3*, and *Apoe* in PrL tissue. ***C***, Left panel, Graph showing average number of *Apoe* transcripts per astrocyte (*Gfap*+ cell) in the PrL. Right panel, Graph showing percentage of astrocytes (*Gfap*+ cells) that coexpress *Apoe* in the PrL. No significant differences in *Apoe* expression in astrocytes were observed between conditions or sexes. ***D***, Left panel, Graph showing average number of *Apoe* transcripts per neuron (*Rbfox3*+ cell) in the PrL. Right panel, Graph showing percentage of neurons (*Rbfox3*+ cells) that coexpress *Apoe* in the PrL. *Apoe* expression significantly increased in neurons following LC depolarization (*F*_(1,84)_ = 15.777, *p* = 0.00015, main effect of sex, *F*_(1,84)_ = 10.498, *p* = 0.00171, main effect of condition for number of *Apoe* transcripts per *Rbfox3 *+ cell, and *F*_(1,84)_ = 40.078, *p* = 1.14 × 10^−8^, main effect of condition for % *Rbfox3*+ cells coexpressing *Apoe*).

We next looked for evidence of *Apoe* expression in astrocytes and neurons in the PrL following chemogenetic stimulation of LC inputs (Fig. 3*B*) and found that neither number of *Apoe* transcripts nor percentage of cells expressing *Apoe* significantly differed between group or sex in astrocytes (*Gfap*-expressing cells; Fig. 3*C*) but that both number of transcripts and percentage of cells expressing *Apoe* were significantly increased in putative neurons (*Rbfox3*-expressing cells; *F*_(1,84)_ = 10.498, *p *= 0.00171, main effect of group for number of *Apoe* transcripts; *F*_(1,84)_ = 40.078, *p *= 1.14 × 10^−8^, main effect of condition for percentage of neurons expressing *Apoe*). Additionally, the number of *Apoe* transcripts expressed in *Rbfox3*-expressing cells was significantly higher in females, compared with males (*F*_(1,84)_ = 15.777, *p *= 0.00015, main effect of sex; Fig. 3*D*).

**Figure 4.**
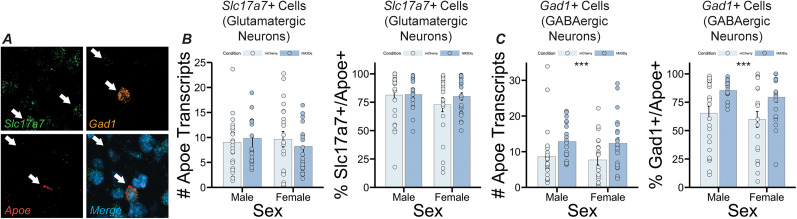
***A***, Enrichment of *Apoe* in GABAergic PrL neurons following stimulation of LC inputs. Confocal image showing expression of *Slc17a7*, *Gad1*, and *Apoe* in PrL tissue. ***B***, Left panel, Graph showing average number of *Apoe* transcripts per glutamatergic neuron (*Slc17a7*+ cell) in the PrL. Right panel, Graph showing percentage of glutamatergic neurons (*Slc17a7*+ cells) that coexpress *Apoe* in the PrL. No significant differences in *Apoe* expression in glutamatergic cells were observed between conditions or sexes. ***C***, Left panel, Graph showing average number of *Apoe* transcripts per GABAergic neuron (*Gad1*+ cell) in the PrL. Right panel, Graph showing percentage of GABAergic neurons (*Gad1*+ cells) that coexpress *Apoe* in the PrL. *Apoe* expression significantly increased in GABAergic neurons following LC depolarization (*F*_(1,84)_ = 9.154, *p* = 0.00329, main effect of condition for number of *Apoe* transcripts per *Gad1*+ cell, *F*_(1,84)_ = 12.898, *p* = 0.000553, main effect of condition for % *Gad1*+ cells coexpressing *Apoe*).

To determine whether neuronal increases in *Apoe* were predominantly in excitatory (glutamatergic) or inhibitory (GABAergic) neurons, we next looked at *Apoe* expression in *Slc17a7*-expressing cells (*Slc17a7* encodes the glutamate transporter protein) and *Gad1*-expressing cells in PrL tissue (Fig. 4*A*). We found that neither number of *Apoe* transcripts nor percentage of cells expressing *Apoe* significantly differed between group or sex in putative excitatory neurons (Fig. 4*B*) but were both significantly increased in putative inhibitory neurons in the experimental group compared with the control group, irrespective of biological sex (*F*_(1,84)_ = 9.154, *p *= 0.00329, main effect of group for number of *Apoe* transcripts; *F*_(1,84)_ = 12.898, *p *= 0.000553, main effect of group for percentage of *Gad1*-expressing neurons coexpressing *Apoe*; Fig. 4*C*).

**Figure 5.**
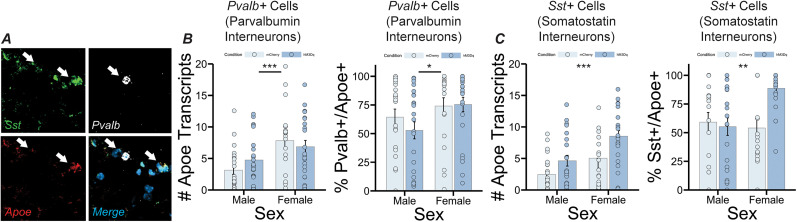
Sex-specific enrichment of *Apoe* in GABAergic neuron subtypes following stimulation of LC inputs. ***A***, Confocal image showing expression of *Pvalb*, *Sst*, and *Apoe* in PrL tissue. ***B***, Left panel, Graph showing average number of *Apoe* transcripts per parvalbumin interneuron (*Pvalb*+ cell) in the PrL. Right panel, Graph showing percentage of parvalbumin interneurons (*Pvalb*+ cells) that coexpress *Apoe* in the PrL. *Apoe* expression in parvalbumin interneurons was significantly higher in females (*F*_(1,86)_ = 12.961, *p* = 0.000531, main effect of sex for number of *Apoe* transcripts per *Pvalb*+ cell, *F*_(1,86)_ = 5.536, *p* = 0.0209, main effect of sex for % *Pvalb*+ cells coexpressing *Apoe*). ***C***, Left panel, Graph showing average number of *Apoe* transcripts per somatostatin interneuron (*Sst*+ cell) in the PrL. Right panel, Graph showing percentage of somatostatin interneurons (*Sst*+ cells) that coexpress *Apoe* in the PrL. *Apoe* expression significantly increased in somatostatin interneurons following LC depolarization (*F*_(1,86)_ = 11.088, *p* = 0.001281, main effect of condition, *F*_(1,86)_ = 13.425, *p* = 0.000429, main effect of sex for number of *Apoe* transcripts per *Sst*+ cell, *F*_(1,86)_ = 7.476, *p* = 0.00759, condition × sex interaction for % *Sst*+ cells coexpressing *Apoe*).

Finally, we looked at *Apoe* expression in two different subtypes of inhibitory neuron—parvalbumin interneurons (*Pvalb*-expressing cells) and somatostatin interneurons (*Sst*-expressing cells; Fig. 5*A*). We found no evidence of increased *Apoe* expression in putative parvalbumin interneurons in the experimental group—however, we did find an increased number of *Apoe* transcripts in *Pvalb*-expressing cells in females compared with males (*F*_(1,86)_ = 12.961, *p *= 0.000531, main effect of sex), as well as an increase in the percentage of *Pvalb*-expressing cells that coexpressed *Apoe* in females compared with males (*F*_(1,86)_ = 5.536, *p *= 0.0209; Fig. 5*B*), suggesting that there are baseline differences in *Apoe* expression in cortical parvalbumin interneurons between sexes in mice. *Apoe* expression was, however, increased in *Sst*-expressing cells in the experimental group (*F*_(1,86)_ = 11.088, *p *= 0.001281, main effect of group for number of *Apoe* transcripts). The increase in *Apoe* expression in putative somatostatin interneurons was partially sex dependent—the number of *Apoe* transcripts in *Sst*-expressing cells was also higher in females compared with males (*F*_(1,86)_ = 13.425, *p *= 0.000429, main effect of sex for number of *Apoe* transcripts), and analysis of the percentage of *Sst*-expressing cells that coexpressed *Apoe* revealed a sex × group interaction (*F*_(1,86)_ = 7.476, *p *= 0.00759), with an increase in the female experimental group specifically.

## Discussion

We find that chemogenetic activation of a circuit that is involved in attention (Hallock et al., 2024) induces cortical expression of genes in mice that could be used for molecular identifiers of function in this circuit. Specifically, we find that one of these genes (*Apoe*) is selectively enriched in GABAergic neurons following circuit activation. These results provide insight into how distinct anatomical inputs might regulate the transcriptome of cortical regions that are involved in a variety of behaviors, such as the prelimbic subregion of the frontal cortex. The design employed in this study compliments research demonstrating behaviorally induced (Cho et al., 2016; Mukherjee et al., 2018) and stimulation-induced (Nelson et al., 2023; Bach et al., 2024) changes in gene expression in rodents. The identification of genes that are upregulated upon activation of brainstem→cortical circuits might serve as a starting point for the development of targeted treatments for cognitive deficits in disorders in which these circuits are dysfunctional, such as ADHD, schizophrenia, and MDD. Interestingly, we also found that a sizable number (82) of genes had reduced transcription in the PrL following stimulation of PrL-projecting neurons in the LC. Gene ontology analysis revealed that these genes are involved in ornithine decarboxylase inhibitor activity, negative regulation of polyamine transmembrane transport, and phosphatase complexes (for a complete list of gene ontology terms and core gene sets, see Extended Data Fig. 1-2). At the individual level, there was no discernable functional relationship between top differentially expressed genes that were downregulated in the CNO group. Some of these genes are implicated in animal models of stress and depressive symptoms (*Gm27177* and *Krt80*, for example; Dai et al., 2022; Lanshakov et al., 2024). Others are involved in olfaction (*Bpifb9a*; Kuntová et al., 2018) and abnormal tau phosphorylation in animal models of Alzheimer's disease (AD; *Hs3st2*; Sepulveda-Diaz et al., 2015). LC efferents modulate inhibitory transmission in cortex (Toussay et al., 2013), and a large number of cortical interneurons express ɑ1, β1, and β2 adrenergic receptors (Papay et al., 2006; Santana et al., 2013), raising the possibility that the decrease in transcription of some genes in our study is due to elevated interneuron activity following LC→PrL stimulation. Although we were not able to verify that this was the case, our observation that *Apoe* transcription increased selectively in interneurons is in line with this hypothesis. Correspondingly, we also did not see increased transcription of any canonical immediate early genes (IEGs), such as *Fos*, *Arc*, or *Npas4*. This raises the possibility that our stimulation protocol was not sufficient to elicit activation of PrL-projecting LC neurons or detect the presence of IEGs in PrL tissue. Several lines of evidence argue against this interpretation, however. First, results shown in Figure 2 demonstrate that *Fos* transcription was heavily increased in these neurons following CNO administration in our DREADD-expressing mice, showing that these neurons were indeed activated with our CNO dose and timeline. Second, previous studies using the same timeline (30 min for CNO and 90 min for IEG expression, 120 min total) and experimental protocol in PrL-projecting hippocampal neurons found robust IEG expression in PrL tissue (Hallock et al., 2020), suggesting that the lack of IEG expression found in the current study is due to differences in the neural circuitry under investigation.

The *APOE* gene encodes for a polymorphic protein that is implicated in processes such as neurogenesis, plasticity, and neuronal repair (Rusted et al., 2013). *APOE* has four allelic variants in humans, with the fourth allele (−e4) being heavily linked to the development of AD. Patients with AD exhibit many facets of attentional and memory deficits; for example, selective attention, or the ability to attend to a particular stimulus in the environment, and executive control of attention, or coordinating goal-directed activities, are both impaired in AD (Parasuraman et al., 2002). *APOE* genotype, even in the absence of AD pathology, modulates a range of processes, including visuospatial attention and working memory (Espeseth et al., 2006). Interestingly, deficits in attention, specifically the shifting of visuospatial attention, have been found in *APOE* −e4 allele carriers who do not have dementia (Greenwood et al., 2000).

*APOE* allele type impacts facets of attention and memory across the lifespan as well; younger individuals who are carriers of the −e4 allele perform better on measures of sustained and covert attention when compared with younger individuals who are not carriers of the −e4 allele (Rusted et al., 2013). Interestingly, advantages in performance noted early in life for −e4 carriers might turn into disadvantages later in life. Filippini et al. (2011) looked at blood oxygen level-dependent (BOLD) signals in both older and younger carriers, and noncarriers, of the −e4 allele and found that older −e4 individuals exhibited significantly increased activation of several brain regions relative to younger −e4 carriers, an effect that was not observed between young and old −e4 carriers. Filippini et al. (2011) proposed that the −e4 allele might impact age-related compensatory processes. Similarly, Jochemsen et al. (2012) found that younger −e4 carriers had better memory recall compared with participants with other *APOE* genotypes, while older −e4 carriers had worse recall. It is possible that the age- and allele-related decline in task performance observed with relation to *APOE* is related to neurophysiological changes, such as an increase in amyloid beta deposition, which is a hallmark of AD (Filippini et al., 2011). The −e4 allele has the least amount of lipidation out of any of the *APOE* alleles (Flowers and Rebeck, 2020), which means that the proteins encoded by the *APOE* −e4 allele are more likely to aggregate and become toxic to neurons.

In order to fully understand the function of *APOE* as it relates to neural circuits that are involved in cognitive aspects of neuropsychiatric and neurodegenerative disorders, such as attention and memory, it is necessary to know where *APOE* is being expressed within those circuits. LC neurons degenerate over the progression of AD and have been identified as one of the first locations in the brain to accumulate tau protein, a pathological hallmark of AD (Dahl et al., 2020; James et al., 2020). Alterations in LC-NE system function have also been implicated in schizophrenia (Suttkus et al., 2021) and ADHD (Ressler and Nemeroff, 2001). Ideally, if *APOE* is used as a potential biomarker for LC→frontal cortex function, it could help shed light on how and when this circuit might malfunction in patients with these disorders. In a healthy human brain, *APOE* is expressed primarily in astrocytes (Flowers and Rebeck, 2020). Astrocytes provide neurons with cholesterol, and *APOE* is the predominant carrier for cholesterol transport to these neurons (D. Li et al., 2022). Similar to humans, *Apoe* is primarily expressed in astrocytes in wild-type rodent brains (Raber et al., 1998). However, Q. Xu et al. (2006) found that upon excitotoxic injury, hippocampal neurons in the mouse brain will express *Apoe*. Q. Xu et al. (2006) also proposed that, depending on where *Apoe* is being expressed, it could serve different functions; for example, in damaged neurons, *Apoe* might be involved in mitochondrial dysfunction and neurofibrillary tangle formation, and in astrocytes, it may be involved in the formation of amyloid plaques. Other papers have demonstrated that *APOE* is expressed in human (P. T. Xu et al., 1999) and rodent (Boschert et al., 1999; Harris et al., 2004) neurons, suggesting that the APOE protein functions in both astrocytes and neurons to regulate circuit function. Elucidating where *Apoe* is being expressed in brain regions that are involved in attention and memory may shed light on what *Apoe*'s role is in those circuits and which metabolic products or molecules related to neuropsychiatric conditions *Apoe* is involved in producing. Our finding that activation of one such circuit induces upregulation of *Apoe* in neurons, and not astrocytes, is a step toward this goal.

We found that there were no statistically significant differences in the average number of *Apoe* transcripts per putative excitatory (*Slc17a7*-expressing) neuron, as well as the proportion of *Slc17a7*-expressing neurons that coexpressed *Apoe* between DREADD-expressing and control mice. These results suggest that excitatory neurons are therefore not responsible for the upregulation of *Apoe* found upon activation of LC→PrL projection neurons. We instead observed that a significantly greater average number of inhibitory neurons coexpressed *Apoe* in DREADD-expressing mice and that the average number of *Apoe* transcripts in *Gad1*-expressing cells was significantly higher in DREADD-expressing mice compared with controls. We therefore conclude that inhibitory neurons are responsible for the upregulation of *Apoe* following depolarization of PrL-projecting neurons in the LC. Inhibitory neurons are involved in sculpting local networks; they mostly lack long range projections and instead influence the activity of local circuitry (Moore, 1993). The major neurotransmitter used by inhibitory neurons is gamma-aminobutyric acid (GABA). In the present study, we used *Gad1* as a general marker for inhibitory neurons. The *Gad1* gene encodes for glutamic acid decarboxylase (GAD), which is an enzyme that catalyzes the decarboxylation of glutamate in GABA synthesis (Bu et al., 1992).

Previous research suggests that there is increased susceptibility of GABAergic interneurons to *APOE* −e4-related pathology (Najm et al., 2019). There exists an interesting connection between *APOE* allele type and GABAergic inhibitory activity, in that *APOE* −e4 has been associated with hyperactivity of brain function, measured through BOLD signals, in young carriers (Filippini et al., 2011). It is possible that this reflects a difference in GABAergic inhibitory neuronal function, especially given the association between *APOE* −e4 and subclinical epileptiform activity that can occur in patients without dementia when experiencing stress (Palop and Mucke, 2009; Andrews-Zwilling et al., 2010). Dysfunction in GABAergic neurons is also a hallmark of many neuropsychiatric disorders, including schizophrenia (Gonzalez-Burgos et al., 2010; Cohen et al., 2015), ADHD (Edden et al., 2012; Ferranti et al., 2024), and MDD (Luscher et al., 2011). At a molecular level, *APOE* −e4 undergoes proteolytic cleavage in neurons, which generates neurotoxic fragments (Brecht et al., 2004). These fragments eventually lead to tau phosphorylation, a key component, alongside amyloid beta plaque formation, of AD (Brecht et al., 2004; Najm et al., 2019). G. Li et al. (2009) found that levels of neurotoxic *Apoe* fragments and tau phosphorylation were elevated in the hippocampal neurons of mice that expressed *APOE* −e4. They also found decreased levels of GABAergic interneuron survival in these mice. This finding demonstrates that GABAergic interneurons, specifically in brain regions like the hippocampus, are susceptible to *APOE* −e4-derived neurotoxic fragments and tau pathology (Andrews-Zwilling et al., 2010). Both factors contribute to decreased survival of GABAergic interneurons, which also leads to learning and memory deficit characteristic of AD (Knoferle et al., 2014).

Because *Gad1* is a general marker for inhibitory neurons, it cannot delineate which subtype of interneuron, if any, is responsible for the upregulation of *Apoe* found in this experiment. To investigate this, we examined *Apoe* expression in two major subtypes of inhibitory neuron: parvalbumin interneurons and somatostatin interneurons. Cortical parvalbumin interneurons mainly provide perisomatic inhibition to excitatory pyramidal neurons, while somatostatin interneurons mainly target pyramidal neuron dendrites (Hangya et al., 2014). We find that enrichment of *Apoe* is primarily observed in putative somatostatin interneurons following activation of PrL-projecting LC neurons. Furthermore, this effect is much stronger in female mice, revealing that cell type-specific expression of *Apoe* is sex dependent. Somatostatin interneurons in the cortex are widely implicated in a range of behaviors that are affected in neuropsychiatric disorders, such as working memory (D. Kim et al., 2016; Abbas et al., 2018), attention (Urban-Ciecko et al., 2018), and learning (Adler et al., 2019). *Apoe* may therefore act as a molecular regulator of function in these neurons during cognitive processing. Interestingly, previous research has demonstrated that the distribution of somatostatin interneurons in several brain regions is sexually dimorphic (Y. Kim et al., 2017). Deficits in somatostatin gene production in the cingulate cortex of patients with MDD is also more pronounced in female patients (Tripp et al., 2011; Seney et al., 2015), indicating that sex differences in somatostatin interneuron function may contribute to differences in the onset and severity of neuropsychiatric disorders between biological males and females. Further research is needed to uncover which interneuron subtypes underlie increases in *Apoe* expression observed in male mice in our study.

Taken together, our findings provide an interesting link between inhibitory neurons and activity-induced *Apoe* expression in the prelimbic subregion of the medial frontal cortex. Although the research surrounding *Apoe* and GABAergic interneurons has traditionally focused on learning and memory deficits and allele-specific effects, it is possible that a connection exists between *Apoe*, GABAergic interneuron function, and cognitive deficits more broadly. Loss of GABAergic interneurons in *APOE* −e4 carriers is associated with learning and memory deficits, but these occur after attentional deficits arise during the progression of AD. Therefore, it is possible that *APOE*-related GABAergic interneuron loss is occurring in brain regions that are involved in attention and memory, such as the PrL and the LC. Our results suggest that cell type-specific regulation of *Apoe* might be useful as a diagnostic biomarker of circuit function in neuropsychiatric and neurodegenerative disorders. A crucial next step would be to examine *APOE* transcription in cingulate cortex interneurons in patients with disorders that present with attention and memory symptoms, such as AD, schizophrenia, and ADHD. Importantly, although the structure of the mouse *Apoe* gene and human *APOE* gene are different, a basic understanding of how and where *Apoe* is expressed in specific circuits is a foundational step toward illuminating how human alleles might impact cellular function to affect cognitive domains such as attention and memory.

## Synthesis

Reviewing Editor: Timothy Jarome, Virginia Polytechnic Institute and State University Decisions are customarily a result of the Reviewing Editor and the peer reviewers coming together and discussing their recommendations until a consensus is reached. When revisions are invited, a fact-based synthesis statement explaining their decision and outlining what is needed to prepare a revision will be listed below. The following reviewer(s) agreed to reveal their identity: NONE.

Both reviewers noted the significant improvement in the manuscript and that concerns raised in the initial review have been addressed. However, the abstract still contains an assertion that Apoe may be a novel target for ameliorating attentional impairments in neuropsychiatric disease. As the rebuttal stated that these assertions were removed, the abstract should be reviewed and corrected as appropriate

## References

[B1] Abbas AI, Sundiang MJ, Henoch B, Morton MP, Bolkan SS, Park AJ, Harris AZ, Kellendonk C, Gordon JA (2018) Somatostatin interneurons facilitate hippocampal-prefrontal synchrony and prefrontal spatial encoding. Neuron 100:926–939. 10.1016/j.neuron.2018.09.029 30318409 PMC6262834

[B2] Adler A, Zhao R, Shin ME, Yasuda R, Gan WB (2019) Somatostatin-expressing interneurons enable and maintain learning-dependent sequential activation of pyramidal neurons. Neuron 102:202–216. 10.1016/j.neuron.2019.01.036 30792151 PMC6555419

[B3] Andrews-Zwilling Y, Bien-Ly N, Xu Q, Li G, Bernardo A, Yoon SY, Zwilling D, Yan T, Chen L, Huang Y (2010) Apolipoprotein E4 causes age-and tau-dependent impairment of GABAergic interneurons, leading to learning and memory deficits in mice. J Neurosci 30:13707–13717. 10.1523/JNEUROSCI.4040-10.2010 20943911 PMC2988475

[B4] Ashburner M, et al. (2000) Gene ontology: tool for the unification of biology. Nat Genet 25:25–29. 10.1038/75556 10802651 PMC3037419

[B5] Bach SV, Bauman AJ, Hosein D, Tuscher JJ, Ianov L, Greathouse KM, Henderson BW, Herskowitz JH, Martinowich K, Day JJ (2024) Distinct roles of Bdnf I and Bdnf IV transcript variant expression in hippocampal neurons. Hippocampus 34:218–229. 10.1002/hipo.23600 38362938 PMC11039386

[B6] Bahtiyar S, Karaca KG, Henckens MJ, Roozendaal B (2020) Norepinephrine and glucocorticoid effects on the brain mechanisms underlying memory accuracy and generalization. Mol Cell Neurosci 108:103537. 10.1016/j.mcn.2020.103537 32805389

[B7] Bangasser DA, Wiersielis KR, Khantsis S (2016) Sex differences in the locus coeruleus-norepinephrine system and its regulation by stress. Brain Res 1641:177–188. 10.1016/j.brainres.2015.11.021 26607253 PMC4875880

[B8] Bangasser DA, Zhang X, Garachh V, Hanhauser E, Valentino RJ (2011) Sexual dimorphism in locus coeruleus dendritic morphology: a structural basis for sex differences in emotional arousal. Physiol Behav 103:342–351. 10.1016/j.physbeh.2011.02.037 21362438 PMC3081983

[B9] Barry DN, Commins S (2017) Temporal dynamics of immediate early gene expression during cellular consolidation of spatial memory. Behav Brain Res 327:44–53. 10.1016/j.bbr.2017.03.019 28330743

[B10] Berridge CW, Spencer RC (2016) Differential cognitive actions of norepinephrine a2 and a1 receptor signaling in the prefrontal cortex. Brain Res 1641:189–196. 10.1016/j.brainres.2015.11.024 26592951 PMC4876052

[B11] Birrell JM, Brown VJ (2000) Medial frontal cortex mediates perceptual attentional set shifting in the rat. J Neurosci 20:4320–4324. 10.1523/JNEUROSCI.20-11-04320.2000 10818167 PMC6772641

[B12] Boschert U, Merlo-Pich E, Higgins G, Roses AD, Catsicas S (1999) Apolipoprotein E expression by neurons surviving excitotoxic stress. Neurobiol Dis 6:508–514. 10.1006/nbdi.1999.0251 10600406

[B13] Bouret S, Sara SJ (2004) Reward expectation, orientation of attention and locus coeruleus-medial frontal cortex interplay during learning. Eur J Neurosci 20:791–802. 10.1111/j.1460-9568.2004.03526.x 15255989

[B14] Brecht WJ, et al. (2004) Neuron-specific apolipoprotein e4 proteolysis is associated with increased tau phosphorylation in brains of transgenic mice. J Neurosci 24:2527–2534. 10.1523/JNEUROSCI.4315-03.2004 15014128 PMC6729489

[B15] Brito GN, Brito LS (1990) Septohippocampal system and the prelimbic sector of frontal cortex: a neuropsychological battery analysis in the rat. Behav Brain Res 36:127–146. 10.1016/0166-4328(90)90167-D 2302312

[B16] Bu DF, Erlander MG, Hitz BC, Tillakaratne NJ, Kaufman DL, Wagner-McPherson CB, Evans GA, Tobin AJ (1992) Two human glutamate decarboxylases, 65-kDa GAD and 67-kDa GAD, are each encoded by a single gene. Proc Natl Acad Sci U S A 89:2115–2119. 10.1073/pnas.89.6.2115 1549570 PMC48607

[B17] Caballero-Puntiverio M, Lerdrup LS, Grupe M, Larsen CW, Dietz AG, Andreasen JT (2019) Effect of ADHD medication in male C57BL/6J mice performing the rodent continuous performance test. Psychopharmacology 236:1839–1851. 10.1007/s00213-019-5167-x 30656365

[B18] Carter CS, Braver TS, Barch DM, Botvinick MM, Noll D, Cohen JD (1998) Anterior cingulate cortex, error detection, and the online monitoring of performance. Science 280:747–749. 10.1126/science.280.5364.747 9563953

[B19] Cho J, Huang BS, Gray JM (2016) RNA sequencing from neural ensembles activated during fear conditioning in the mouse temporal association cortex. Sci Rep 6:31753. 10.1038/srep31753 27557751 PMC4997356

[B20] Cohen SM, Tsien RW, Goff DC, Halassa MM (2015) The impact of NMDA receptor hypofunction on GABAergic neurons in the pathophysiology of schizophrenia. Schizophr Res 167:98–107. 10.1016/j.schres.2014.12.026 25583246 PMC4724170

[B21] Dahl MJ, Mather M, Sander MC, Werkle-Bergner M (2020) Noradrenergic responsiveness supports selective attention across the adult lifespan. J Neurosci 40:4372–4390. 10.1523/JNEUROSCI.0398-19.2020 32317388 PMC7252473

[B22] Dai W, Huang S, Luo Y, Cheng X, Xia P, Yang M, Ye X (2022) Sex-specific transcriptomic signatures in brain regions critical for neuropathic pain-induced depression. Front Mol Neurosci 15:886916. 10.3389/fnmol.2022.886916 35663269 PMC9159910

[B23] Darmanis S, Sloan SA, Zhang Y, Enge M, Caneda C, Shuer LM, Hayden-Gephart MG, Quake SR (2015) A survey of human brain transcriptome diversity at the single cell level. Proc Natl Acad Sci U S A 112:7285–7290. 10.1073/pnas.1507125112 26060301 PMC4466750

[B24] Davis KD, Hutchison WD, Lozano AM, Tasker RR, Dostrovsky JO (2000) Human anterior cingulate cortex neurons modulated by attention-demanding tasks. J Neurophysiol 83:3575–3577. 10.1152/jn.2000.83.6.3575 10848573

[B25] Delatour B, Gisquet-Verrier P (1996) Prelimbic cortex specific lesions disrupt delayed-variable response tasks in the rat. Behav Neurosci 110:1282. 10.1037/0735-7044.110.6.1282 8986332

[B26] del Cerro I, et al. (2020) Locus coeruleus connectivity alterations in late-life major depressive disorder during a visual oddball task. Neuroimage Clin 28:102482. 10.1016/j.nicl.2020.102482 33371943 PMC7649653

[B27] Edden RA, Crocetti D, Zhu H, Gilbert DL, Mostofsky SH (2012) Reduced GABA concentration in attention-deficit/hyperactivity disorder. Arch Gen Psychiatry 69:750–753. 10.1001/archgenpsychiatry.2011.2280 22752239 PMC3970207

[B28] Espeseth T, Greenwood PM, Reinvang I, Fjell AM, Walhovd KB, Westlye LT, Wehling E, Lundervold A, Rootwelt H, Parasuraman R (2006) Interactive effects of APOE and CHRNA4 on attention and white matter volume in healthy middle-aged and older adults. Cogn Affect Behav Neurosci 6:31–43. 10.3758/CABN.6.1.31 16869227

[B29] Ferranti AS, Luessen DJ, Niswender CM (2024) Novel pharmacological targets for GABAergic dysfunction in ADHD. Neuropharmacology 249:109897. 10.1016/j.neuropharm.2024.109897 38462041 PMC11843668

[B30] Filippini N, Ebmeier KP, MacIntosh BJ, Trachtenberg AJ, Frisoni GB, Wilcock GK, Beckmann CF, Smith SM, Matthews PM, Mackay CE (2011) Differential effects of the APOE genotype on brain function across the lifespan. Neuroimage 54:602–610. 10.1016/j.neuroimage.2010.08.009 20705142

[B31] Fisher BM, Saksida LM, Robbins TW, Bussey TJ (2020) Functional dissociations between subregions of the medial prefrontal cortex on the rodent touchscreen continuous performance test (rCPT) of attention. Behav Neurosci 134:1–14. 10.1037/bne0000338 31829644

[B32] Flowers SA, Rebeck GW (2020) APOE in the normal brain. Neurobiol Dis 136:104724. 10.1016/j.nbd.2019.104724 31911114 PMC7002287

[B33] Giustino TF, Fitzgerald PJ, Ressler RL, Maren S (2019) Locus coeruleus toggles reciprocal prefrontal firing to reinstate fear. Proc Natl Acad Sci U S A 116:8570–8575. 10.1073/pnas.1814278116 30971490 PMC6486780

[B34] Gonzalez-Burgos G, Hashimoto T, Lewis DA (2010) Alterations of cortical GABA neurons and network oscillations in schizophrenia. Curr Psychiatry Rep 12:335–344. 10.1007/s11920-010-0124-8 20556669 PMC2919752

[B35] Greenwood PM, Sunderland T, Friz JL, Parasuraman R (2000) Genetics and visual attention: selective deficits in healthy adult carriers of the ɛ4 allele of the apolipoprotein E gene. Proc Natl Acad Sci U S A 97:11661–11666. 10.1073/pnas.97.21.11661 11027364 PMC17257

[B36] Hallock HL, Adiraju SS, Miranda-Barrientos J, McInerney JM, Oh S, DeBrosse AC, Li Y, Carr GV, Martinowich K (2024) Electrophysiological correlates of attention in the locus coeruleus-prelimbic cortex circuit during the rodent continuous performance test. Neuropsychopharmacology 49:521–531. 10.1038/s41386-023-01692-3 37563281 PMC10789747

[B37] Hallock HL, Quillian HM 4th, Maynard KR, Mai Y, Chen HY, Hamersky GR, Martinowich K (2020) Molecularly defined hippocampal inputs regulate population dynamics in the prelimbic cortex to suppress context fear memory retrieval. Biol Psychiatry 88:554–565. 10.1016/j.biopsych.2020.04.014 32560963 PMC7487039

[B38] Hallock HL, Wang A, Griffin AL (2016) Ventral midline thalamus is critical for hippocampal–prefrontal synchrony and spatial working memory. J Neurosci 36:8372–8389. 10.1523/JNEUROSCI.0991-16.2016 27511010 PMC4978800

[B39] Hangya B, Pi HJ, Kvitsiani D, Ranade SP, Kepecs A (2014) From circuit motifs to computations: mapping the behavioral repertoire of cortical interneurons. Curr Opin Neurobiol 26:117–124. 10.1016/j.conb.2014.01.007 24508565 PMC4090079

[B40] Harris FM, Tesseur I, Brecht WJ, Xu Q, Mullendorff K, Chang S, Wyss-Coray T, Mahley RW, Huang Y (2004) Astroglial regulation of apolipoprotein E expression in neuronal cells: implications for Alzheimer's disease. J Biol Chem 279:3862–3868. 10.1074/jbc.M309475200 14585838

[B41] James T, Kula B, Choi S, Khan SS, Bekar LK, Smith NA (2020) Locus coeruleus in memory formation and Alzheimer’s disease. Eur J Neurosci 54:6948–6959. 10.1111/ejn.15045 33190318 PMC8121900

[B42] Jochemsen HM, Muller M, van der Graaf Y, Geerlings MI (2012) APOE ε4 differentially influences change in memory performance depending on age. the SMART-MR study. Neurobiol Aging 33:832.e15. 10.1016/j.neurobiolaging.2011.07.016 21908077

[B43] Jodoj E, Chiang C, Aston-Jones G (1998) Potent excitatory influence of prefrontal cortex activity on noradrenergic locus coeruleus neurons. Neuroscience 83:63–79. 10.1016/S0306-4522(97)00372-2 9466399

[B44] Joshi S, Gold JI (2022) Context-dependent relationships between locus coeruleus firing patterns and coordinated neural activity in the anterior cingulate cortex. Elife 11:e63490. 10.7554/eLife.63490 34994344 PMC8765756

[B45] Kerns JG, Cohen JD, MacDonald AW, Johnson MK, Stenger VA, Aizenstein H, Carter CS (2005) Decreased conflict- and error-related activity in the anterior cingulate cortex in subjects with schizophrenia. Am J Psychiatry 162:1833–1839. 10.1176/appi.ajp.162.10.1833 16199829

[B46] Kim Y, et al. (2017) Brain-wide maps reveal stereotyped cell-type-based cortical architecture and subcortical sexual dimorphism. Cell 171:456–469. 10.1016/j.cell.2017.09.020 28985566 PMC5870827

[B47] Kim D, Jeong H, Lee J, Ghim JW, Her ES, Lee SH, Jung MW (2016) Distinct roles of parvalbumin-and somatostatin-expressing interneurons in working memory. Neuron 92:902–915. 10.1016/j.neuron.2016.09.023 27746132

[B48] Kim D, Langmead B, Salzberg SL (2015) HISAT: a fast spliced aligner with low memory requirements. Nat Methods 12:357–360. 10.1038/nmeth.3317 25751142 PMC4655817

[B49] Knoferle J, Yoon SY, Walker D, Leung L, Gillespie AK, Tong LM, Bien-Ly N, Huang Y (2014) Apolipoprotein E4 produced in GABAergic interneurons causes learning and memory deficits in mice. J Neurosci 34:14069–14078. 10.1523/JNEUROSCI.2281-14.2014 25319703 PMC4198545

[B50] Kozlovskiy SA, Nikonova EY, Pyasik MM, Velichkovsky BM (2012) The cingulate cortex and human memory processes. Psychol Russ 5:231. 10.11621/pir.2012.0014

[B51] Kuntová B, Stopková R, Stopka P (2018) Transcriptomic and proteomic profiling revealed high proportions of odorant binding and antimicrobial defense proteins in olfactory tissues of the house mouse. Front Genet 9:26. 10.3389/fgene.2018.00026 29459883 PMC5807349

[B52] Lanshakov DA, Sukhareva EV, Bulygina VV, Khozyainova AA, Gerashchenko TS, Denisov EV, Kalinina TS (2024) Brainstem transcriptomic changes in male Wistar rats after acute stress, comparing the use of duplex specific nuclease (DSN). Sci Rep 14:21856. 10.1038/s41598-024-73042-2 39300279 PMC11412992

[B53] Laubach M, Amarante LM, Swanson K, White SR (2018) What, if anything, is rodent prefrontal cortex? eNeuro 5:1–14. 10.1523/ENEURO.0315-18.2018 30406193 PMC6220587

[B54] Law CW, Chen Y, Shi W, Smyth GK (2014) Voom: precision weights unlock linear model analysis tools for RNA-seq read counts. Genome Biol 15:R29. 10.1186/gb-2014-15-2-r29 24485249 PMC4053721

[B55] Lenartowicz A, McIntosh AR (2005) The role of anterior cingulate cortex in working memory is shaped by functional connectivity. J Cogn Neurosci 17:1026–1042. 10.1162/0898929054475127 16102235

[B56] Li G, Bien-Ly N, Andrews-Zwilling Y, Xu Q, Bernardo A, Ring K, Halabisky B, Deng C, Mahley RW, Huang Y (2009) GABAergic interneuron dysfunction impairs hippocampal neurogenesis in adult apolipoprotein E4 knockin mice. Cell Stem Cell 5:634–645. 10.1016/j.stem.2009.10.015 19951691 PMC2992822

[B57] Li D, Zhang J, Liu Q (2022) Brain cell type-specific cholesterol metabolism and implications for learning and memory. Trends Neurosci 45:401–414. 10.1016/j.tins.2022.01.002 35184896

[B58] Luscher B, Shen Q, Sahir N (2011) The GABAergic deficit hypothesis of major depressive disorder. Mol Psychiatry 16:383–406. 10.1038/mp.2010.120 21079608 PMC3412149

[B59] MacDonald AW, Cohen JD, Stenger VA, Carter CS (2000) Dissociating the role of the dorsolateral prefrontal and anterior cingulate cortex in cognitive control. Science 288:1835–1838. 10.1126/science.288.5472.1835 10846167

[B60] Mar AC, Nilsson SRO, Gamallo-Lana B, Lei M, Dourado T, Alsio J, Saksida LM, Bussey TJ, Robbins TW (2017) MAM-E17 rat model impairments on a novel continuous performance task: effects of potential cognitive enhancing drugs. Psychopharmacology 234:2837–2857. 10.1007/s00213-017-4679-5 28744563 PMC5591806

[B61] Maynard KR, et al. (2021) Transcriptome-scale spatial gene expression in the human dorsolateral prefrontal cortex. Nat Neurosci 24:425–436. 10.1038/s41593-020-00787-0 33558695 PMC8095368

[B62] Maynard KR, Tippani M, Takahashi Y, Phan BN, Hyde TM, Jaffe AE, Martinowich K (2020) Dotdotdot: an automated approach to quantify multiplex single molecule fluorescent in situ hybridization (smFISH) images in complex tissues. Nucleic Acids Res 48:e66. 10.1093/nar/gkaa312 32383753 PMC7293004

[B63] McDonald RJ, Hong NS, Atwood A, Tyndall AV, Kolb B (2021) An assessment of the functional effects of amphetamine-induced dendritic changes in the nucleus accumbens, medial prefrontal cortex, and hippocampus on different types of learning and memory function. Neurobiol Learn Mem 180:107408. 10.1016/j.nlm.2021.107408 33609742

[B64] Moore RY (1993) Principles of synaptic transmission. Ann N Y Acad Sci 695:1–9. 10.1111/j.1749-6632.1993.tb23018.x 7902053

[B65] Mukherjee D, et al. (2018) Salient experiences are represented by unique transcriptional signatures in the mouse brain. Elife 7:7. 10.7554/eLife.31220 29412137 PMC5862526

[B66] Murchison CF, Zhang XY, Zhang WP, Ouyang M, Lee A, Thomas SA (2004) A distinct role for norepinephrine in memory retrieval. Cell 117:131–143. 10.1016/S0092-8674(04)00259-4 15066288

[B67] Najm R, Jones EA, Huang Y (2019) Apolipoprotein E4, inhibitory network dysfunction, and Alzheimer’s disease. Mol Neurodegener 14:1–13. 10.1186/s13024-019-0324-6 31186040 PMC6558779

[B68] Nelson ED, Maynard KR, Nicholas KR, Tran MN, Divecha HR, Collado-Torres L, Hicks SC, Martinowich K (2023) Activity-regulated gene expression across cell types of the mouse hippocampus. Hippocampus 33:1009–1027. 10.1002/hipo.23548 37226416 PMC11129873

[B69] Newman LA, Darling J, McGaughy J (2008) Atomoxetine reverses attentional deficits produced by noradrenergic deafferentation of medial prefrontal cortex. Psychopharmacology 200:39–50. 10.1007/s00213-008-1097-8 18568443 PMC10719959

[B70] Palop JJ, Mucke L (2009) Epilepsy and cognitive impairments in Alzheimer disease. Arch Neurol 66:435–440. 10.1001/archneurol.2009.15 19204149 PMC2812914

[B71] Papay R, Gaivin R, Jha A, Mccune DF, Mcgrath JC, Rodrigo MC, Perez DM (2006) Localization of the mouse α1A-adrenergic receptor (AR) in the brain: α1AAR is expressed in neurons, GABAergic interneurons, and NG2 oligodendrocyte progenitors. J Comp Neurol 497:209–222. 10.1002/cne.20992 16705673

[B72] Parasuraman R, Greenwood PM, Sunderland T (2002) The apolipoprotein E gene, attention, and brain function. Neuropsychology 16:254–274. 10.1037/0894-4105.16.2.254 11949718 PMC1350934

[B73] Passetti F, Chudasama Y, Robbins TW (2002) The frontal cortex of the rat and visual attentional performance: dissociable functions of distinct medial prefrontal subregions. Cereb Cortex 12:1254–1268. 10.1093/cercor/12.12.1254 12427677

[B74] Paxinos G, Franklin KB (2019) *Paxinos and Franklin's the mouse brain in stereotaxic coordinates*. London, UK: Academic Press.

[B75] Petersen SE, Fox PT, Posner MI, Mintun M, Raichle ME (1988) Positron emission tomographic studies of the cortical anatomy of single-word processing. Nature 331:585–589. 10.1038/331585a0 3277066

[B76] Pudovkina OL, Kawahara Y, de Vries J, Westerink BH (2001) The release of noradrenaline in the locus coeruleus and prefrontal cortex studied with dual-probe microdialysis. Brain Res 906:38–45. 10.1016/S0006-8993(01)02553-7 11430860

[B77] Raber J, Wong D, Buttini M, Orth M, Bellosta S, Pitas RE, Mahley RW, Lennart-Mucke L (1998) Isoform-specific effects of human apolipoprotein E on brain function revealed in ApoE knockout mice: increased susceptibility of females. Proc Natl Acad Sci U S A 95:10914–10919. 10.1073/pnas.95.18.10914 9724804 PMC27995

[B78] Ressler KJ, Nemeroff CB (2001) Role of norepinephrine in the pathophysiology of neuropsychiatric disorders. CNS Spectr 6:663–670. 10.1017/S1092852900001358 15520614

[B79] Ritchie ME, Phipson B, Wu D, Hu Y, Law CW, Shi W, Smyth GK (2015) Limma powers differential expression analyses for RNA-sequencing and microarray studies. Nucleic Acids Res 43:e47. 10.1093/nar/gkv007 25605792 PMC4402510

[B80] Rusted JM, Evans SL, King SL, Dowell N, Tabet N, Tofts PS (2013) APOE e4 polymorphism in young adults is associated with improved attention and indexed by distinct neural signatures. Neuroimage 65:364–373. 10.1016/j.neuroimage.2012.10.010 23063453

[B81] Santana N, Mengod G, Artigas F (2013) Expression of α1-adrenergic receptors in rat prefrontal cortex: cellular co-localization with 5-HT2A receptors. Int J Neuropsychopharmacol 16:1139–1151. 10.1017/S1461145712001083 23195622

[B82] Seney ML, Tripp A, McCune S, Lewis DA, Sibille E (2015) Laminar and cellular analyses of reduced somatostatin gene expression in the subgenual anterior cingulate cortex in major depression. Neurobiol Dis 73:213–219. 10.1016/j.nbd.2014.10.005 25315685 PMC4394026

[B83] Sepulveda-Diaz JE, Alavi Naini SM, Huynh MB, Ouidja MO, Yanicostas C, Chantepie S, Papy-Garcia D (2015) HS3ST2 expression is critical for the abnormal phosphorylation of tau in Alzheimer’s disease-related tau pathology. Brain 138:1339–1354. 10.1093/brain/awv056 25842390 PMC5963411

[B84] Sherrill LK, Stanis JJ, Gulley JM (2013) Age-dependent effects of repeated amphetamine exposure on working memory in rats. Behav Brain Res 242:84–94. 10.1016/j.bbr.2012.12.044 23291159 PMC3566264

[B85] Shirama A, Takeda T, Ohta H, Iwanami A, Toda S, Kato N (2020) Atypical alert state control in adult patients with ADHD: a pupillometry study. PLoS One 15:e0244662. 10.1371/journal.pone.0244662 33378354 PMC7773233

[B86] Suttkus S, Schumann A, de la Cruz F, Bär KJ (2021) Working memory in schizophrenia: the role of the locus coeruleus and its relation to functional brain networks. Brain Behav 11:e02130. 10.1002/brb3.2130 33784023 PMC8119871

[B87] Toussay X, Basu K, Lacoste B, Hamel E (2013) Locus coeruleus stimulation recruits a broad cortical neuronal network and increases cortical perfusion. J Neurosci 33:3390–3401. 10.1523/JNEUROSCI.3346-12.2013 23426667 PMC6619527

[B88] Tripp A, Kota RS, Lewis DA, Sibille E (2011) Reduced somatostatin in subgenual anterior cingulate cortex in major depression. Neurobiol Dis 42:116–124. 10.1016/j.nbd.2011.01.014 21232602 PMC3039077

[B89] Tronel S, Feenstra MG, Sara SJ (2004) Noradrenergic action in prefrontal cortex in the late stage of memory consolidation. Learn Mem 11:453–458. 10.1101/lm.74504 15254217 PMC498332

[B90] Urban-Ciecko J, Jouhanneau JS, Myal SE, Poulet JF, Barth AL (2018) Precisely timed nicotinic activation drives SST inhibition in neocortical circuits. Neuron 97:611–625. 10.1016/j.neuron.2018.01.037 29420933 PMC6588401

[B91] van Heukelum S, Mars RB, Guthrie M, Buitelaar JK, Beckmann CF, Tiesinga PH, Vogt BA, Glennon JC, Havenith MN (2020) Where is cingulate cortex? A cross-species view. Trends Neurosci 43:285–299. 10.1016/j.tins.2020.03.007 32353333

[B92] Walton ME, Mars RB (2007) Probing human and monkey anterior cingulate cortex in variable environments. Cogn Affect Behav Neurosci 7:413–422. 10.3758/CABN.7.4.413 18189014 PMC2519031

[B93] Xu Q, Bernardo A, Walker D, Kanegawa T, Mahley RW, Huang Y (2006) Profile and regulation of apolipoprotein E (ApoE) expression in the CNS in mice with targeting of green fluorescent protein gene to the ApoE locus. J Neurosci 26:4985–4994. 10.1523/JNEUROSCI.5476-05.2006 16687490 PMC6674234

[B94] Xu PT, Gilbert JR, Qiu HL, Ervin J, Rothrock-Christian TR, Hulette C, Schmechel DE (1999) Specific regional transcription of apolipoprotein E in human brain neurons. Am J Pathol 154:601–611. 10.1016/S0002-9440(10)65305-9 10027417 PMC1850012

[B95] Ye X, Kapeller-Libermann D, Travaglia A, Inda MC, Alberini CM (2017) Direct dorsal hippocampal–prelimbic cortex connections strengthen fear memories. Nat Neurosci 201:52–61. 10.1038/nn.4443 27869801 PMC5191950

[B96] Yu G, Wang LG, Han Y, He QY (2012) Clusterprofiler: an R package for comparing biological themes among gene clusters. OMICS 16:284–287. 10.1089/omi.2011.0118 22455463 PMC3339379

[B97] Zeisel A, et al. (2015) Cell types in the mouse cortex and hippocampus revealed by single-cell RNA-seq. Science 347:1138–1142. 10.1126/science.aaa1934 25700174

